# Polarization induced *Z*_2_ and Chern topological phases in a periodically driving field

**DOI:** 10.1038/srep22993

**Published:** 2016-03-11

**Authors:** Shu-Ting Pi, Sergey Savrasov

**Affiliations:** 1 Department of Physics, University of California, Davis, One Shields Avenus, Davis, California 95616 USA

## Abstract

*Z*_2_ and Chern topological phases such as newly discovered quantum spin Hall and original quantum Hall states hardly both co–exist in a single material due to their contradictory requirement on the time–reversal symmetry (TRS). We show that although the TRS is broken in systems with a periodically driving field, an effective TRS can still be defined provided the ac–field is linearly polarized or certain other conditions are satisfied. The controllable TRS provides us a route to manipulate contradictory phases by tuning the polarization. To demonstrate the idea, we consider a tight-binding model that is relevant to several monolayered materials as a benchmark system. Our calculation shows not only topological *Z*_2_ to Chern phase transition occurs but rich Chern phases are also observed. In addition, we also discussed the realization of our proposal in real materials, such as spin-orbit coupled graphene and crystal Bismuth. This opens the possibility of manipulating various topological phases in a single material and can be a promising approach to engineer new electronic states of matter.

The discovery of topological insulators (TIs) in condensed matter systems has not only revealed novel physics of the quantum world but also unified many physical phenomena, which were thought to be irreverent, into the same framework^1^. Their peculiar edge states make TIs a hot topic for both fundamental interests and industrial applications. Several materials such as HgTe/CdTe quantum well, Bi_*x*_Sb_1−*x*_ alloys, Bi_2_Se_3_ and Bi_2_Te_3_, etc., have been proven to be TIs by experiments^2^,^3^,^4^,^5^,^6^. Despite these successes, how to design a topologically non–trivial material, remains a challenging issue. In most cases, the discovery of new TIs still relies on serendipity rather than predetermination.

Instead of searching for materials with intrinsically non–trivial topology, there are several recent studies focusing on manipulation of topological phases using controllable physical processes, e.g. electric fields, strains, etc^7^,^8^,^9^. Those studies not only offer new tools to generate various topological phases but also open new ways to making real electronic devices.

One of the promising methods to engineer a topological property of a system is to use periodically driving fields^10^,^11^,^12^,^13^,^14^. The proposal is based on the Floquet theory which states that the Hamiltonian of a system with a time–dependent periodic potential can be mapped into an effective static Hamiltonian, called the Floquet Hamiltonian. If the (quasi) band structure of a Floquet Hamiltonian exhibits a topological behavior, we can expect there exists a similar feature in the original Hamiltonian in a dynamical fashion. An advantage of using this method to engineer the band topology is that the ac–field provides a set of tunable parameters such that a variety of band structures unaccessible in the original material can be generated in a dynamical way. Many proposals based on the topology of Floquet Hamiltonians have appeared recently, some of which are: Floquet TIs in graphene^15^,^16^,^17^, Floquet TIs in semiconductor quantum wells^18^, Floquet Majorana fermions in topological superconductors^19^, merging Floquet Dirac points^20^, Floquet fractional Chern insulators^21^, Floquet Weyl semimetal^22^, etc. A few experiments that support the idea of Floquet TIs have also been carried out^23^,^24^. Those works not only lighten up the road to manipulate topological phases but also bring us a vast landscape of new physical phenomena that are hardly found in static systems.

While many topological phases have been studied within the Floquet framework, the discussion of *Z*_2_ phases remains scarce because time–reversal symmetry (TRS), a necessary condition for the existence of the *Z*_2_ phase, is always broken due to the time dependence of the external perturbation. However, the Floquet Hamiltonian is merely an effective mapping of the original Hamiltonian, so the loss of TRS in original Hamiltonian does not necessarily result in the loss of TRS in Floquet Hamiltonian. Establishing an operator that links Floquet states in the Brillouin zone by a similar way as conventional TR operator does, an effective TRS can be defined^11^,^18^. If so, two seemingly contradictory phases, TRS protected *Z*_2_ phase, such as recently discovered quantum spin Hall state, and TRS broken Chern phase, such as much celebrated original quantum Hall state, can both be manipulated in a single material by tuning the ac–field, which is the main message of the present work.

Here, we first show how we truncate the Floquet Hamiltoian to finite dimension in a realistic calculation. Second, we show that the TRS conditions can be easily satisfied if the field is linearly polarized or certain low excitation conditions are reached. Third, we use a prototypical 2D material with strong spin–orbit coupling as a benchmark in our calculation, in order to demonstrate the idea of manipulating *Z*_2_ and Chern topological phases in the same system. More specifically, we consider a generic *p*–orbital honeycomb lattice model to illustrate our findings. Our results show the evidence for rich topological phase transitions among normal phase, *Z*_2_ phase and Chern phases by properly tailoring the external field. In addition, we also found polarization plays as a crucial role in engineering Chern phases. Complex Chern phase diagrams can be solely controlled by the polarization.

## Floquet Theorem

We consider a tight–binding Hamiltonian with an external time perodical ac–field


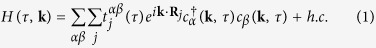


where *τ* is time, **R**_*j*_ the lattice vectors and (*α*, *β*) the internal degrees of freedom (e.g. orbitals, spins, etc.). The ac–field is coupled to the problem by introducing a minimal coupling 
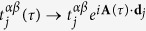
 where **d**_*j*_ is the position vector of state *β* and **A**(*τ*) is the vector potential of the field. Since the Hamiltonian has both lattice and time translational symmetries, we can use Floquet technique to perform a dual Fourier transformation and define an effective static Hamiltonian^20^:


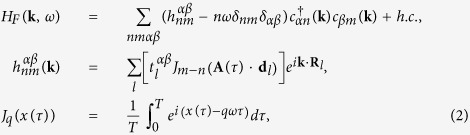


where *ω* = 2*π*/*T* is the frequency of the ac–field and (*n*, *m*) are the Floquet indexes.

The Floquet Hamiltonian *H*_*F*_ forms an eigenvalue problem 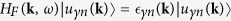 where *γ* is the band index, *n* is the Floquet index ranging −∞ to +∞ and 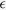
_*γn*_ is the so called quasienergy. The relations 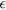
_*γn*_ = 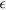
_*γ*0_ + *nω* and 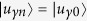 are held as a result of the analogous properties of the Brillouin zone in the frequency domain. They also show the physics of absorbing/emitting *n* photons, so the Floquet bands are shifted by ±*nω*. The solution of the original Hamiltonian is obtained by linearly combining static Floquet band states 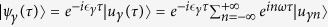 where 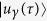 is the Floquet state which is periodic both in space and time. Note that *τ* no longer appears in *H*_*F*_ and 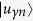, so the Floquet theorem simplifies the original time–dependent problem by mapping it to a static one. Therefore we can treat *H*_*F*_ as the usual lattice Hamiltonian and explore its topology using the techniques developed for static systems. If *H*_*F*_ has non–trival edge states, we can expect a dynamical analogy on 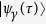
18.

Because the Floquet index *n* ranges from −∞ to +∞, the Floquet Hamiltonian is not manageable unless we make some approximations^10^. Two approximations are frequently adopted: (a) weak intensity limit and (b) high–frequency limit.

**Figure 1:**
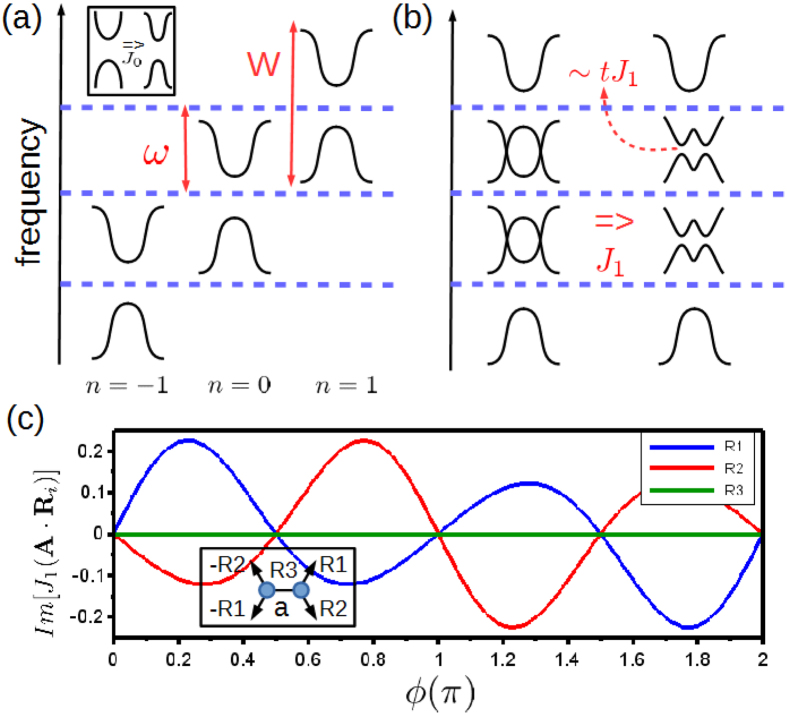
(**a**) Floquet bands within first order emission/absorption photon processes as replicas of the original band structure modified by *J*_0_ (see left upper inset). (**b**) formation of the Floquet band structure by merging states into single Brillouin zone (left) and accounting for the effect of gap opening due to *J*_1_ (right). (**c**) The imaginary part of *J*_1_(**A** · **R**_*j*_) as a function of polarization *ϕ* (in unit of *π*) when **A** = [1, 7.467]/*a* (with *ħ* = *e* = 1). Left lower inset gives the definition of each **R**_*i*_ of graphene honeycomb lattice, where *a* is the length of **R**_3_.

For the approximation (a), let us consider an ac–field sinusoidal in time. In this case, *J*_*q*_(**A** · **d**_*j*_) is essentially the *q*–th Bessel function of the first kind. In the limit of the weak intensity, 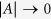, its asymptotic behavior is as follows: *J*_0_ → 1, *J*_*q*≠0_ → 0. The larger the *q* the faster *J*_*q*≠0_ drops to zero. Hence we can truncate *H*_*F*_ to a finite dimension by including just a few lowest order photon processes, provided the field intensity is weak enough. For example, if we keep *q* = 0, 1, *H*_*F*_ is reduced to the following form


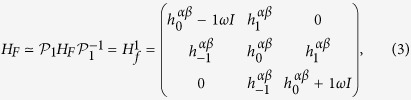


where 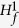 denotes a reduced Floquet Hamiltonian describing an emission/absorbtion of a single photon and 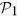 is the operator that projects *H*_*F*_ to 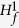. A diagrammatic explanation of such first order process is shown in Fig. 1(a,b). In the upper left inset, the undriven band structure is modified by the 0–th order effect *J*_0_. Once *J*_1_ term comes in, the bands will have three copies with energy shifts 0, ±*ω*. When those bands reach resonant energies, i.e. the band crossings, *J*_1_ will open gaps ~*tJ*_1_ making them anti–crossing. This is the main idea of the truncation.

As for the approximation (b), let us assume the frequency of the external field is so much larger than the bandwidth, 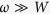, that the Floquet bands do not cross anymore. In this limit, the gap openings due to *J*_*q*>0_ become less important, which implies that it is also the condition to consider just the lowest order photon processes.

## Time-Reversal Symmetry

In an undriven system, the TRS is defined by 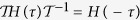 where 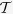 is the conventional TR operator 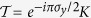. Although systems with time–dependent ac–fields do not hold this property, it is still possible to define an effective TRS for the Floquet Hamiltonian^11^,^18^. To give specific conditions holding the effective TRS, we conclude with two theorems here (see Supplemental Materials):

**Theorem I:** If there exists a parameter *τ*_0_ such that 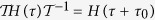, one can always define an effective TR operator 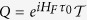 that satisfies the relation 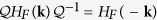.

**Theorem II:** Assuming a system has TRS when it is undriven, i.e. 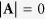, then 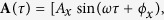 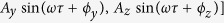 with *ϕ*_*i*_ − *ϕ*_*j*_ = *mπ* (*i*, *j* ∈ *x*, *y*, *z*; *m* ∈ *integers*) will automatically make *H*(*τ*) satisfy 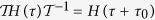. Furthermore if the time frame is properly chosen, one can always let all 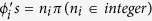 such that *τ*_0_ = 0 and 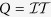.

These theorems tell us if the phase differences among each field component are multiples of *π*, the Floquet Hamiltonian will have effective TRS^22^ and the TR operator can be treated as a conventional one acting in the Hilbert space of the basis of the Floquet Hamiltonian 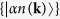. In the following, we will call the condition *ϕ*_*i*_ − *ϕ*_*j*_ = *mπ* as linear polarization in all cases.

The linear polarization condition is not the only option to have effective TRS. Since we are handling the *v*–th order reduced Floquet Hamiltonian 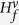 rather than the original *H*_*F*_ in a realistic calculation, it is possible that 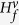 has more time–reversal points than *H*_*F*_. To show this, let us consider the linear polarization case where all polarization angles *ϕ*_*i*_ = 0. In this regard, the integral function *J*_*v*_ is essentially the *v*-th Bessel function of first kind which is a real number for arbitrary *v*. If the Floquet TR condition is held, the Floquet TR operator can be treated as conventional TR operator acting in the Floquet Hilbert space as stated in Theorem II and the matrix elements of *v*-th order Floquet Hamiltonian must satisfy the TRS condition 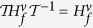, i.e.
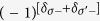
 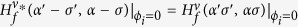, where *σ* = +, − is the spin index and *α* is all the other indies to label a Floquet state. Recall that the hopping integrals in the Floquet Hamiltonian are generated by modifying 
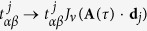
. Such a modification only applies to the orbital part and is irrelevant to the spin degree of freedom. Hence, when 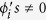, the matrix element can be expressed as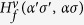 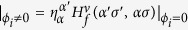 where 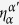 is a complex constant generated via the modification of 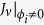. Apparently, if one can properly tailor **A** such that 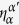 are all real numbers, the above TR condition will still hold even if *ϕ*_*i*_ ≠ 0. It can be considered as an “accidental TRS” which occurs due to a numerical coincidence. It does not exist for arbitrary order in general. However, if we only focus on a few lowest orders, it is possible to find **A** that makes *J*_*q*≤*v*_ real for other polarization angles.

To give an example, we have plotted in Fig. 1(c) the imaginary part of *J*_1_ (*J*_0_ is always real) with respect to three non–equivalent position vectors of a honeycomb lattice as a function of polarization *ϕ* = *ϕ*_*x*_ − *ϕ*_*y*_ by fixing [*A*_*x*_, *A*_*y*_] = [1, 7.467]/*a*. One can immediately notice that there are two additional TRS points (all lines reach 0) other than *ϕ* = *mπ*, i.e. *ϕ* = *π*/2 and *ϕ* = 3*π*/2. These additional TRS are not robust and will be broken for high orders or various amplitudes, so one should confirm that the energy splitting on Kramers degeneracy Δ*E* due to higher order terms is much smaller than the characteristic energy *ε*_*c*_ that we are interested in 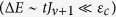 to explore this feature further.

## Floquet Topological Phases

The best candidates to realize topological phase transitions using periodical ac-field would be 2D materials with spin–orbit coupling (SOC), e.g. transition–metal–dichalcogenides, spin-orbit coupled graphene systems, silicene, germanane, etc. These materials have been proven (or have high expectancy) to exhibit monolayer structures with band gaps around dozens to hundreds meV. Some of them are also considered as possible materials to realize Floquet topological phases^15^,^16^,^17^,^25^,^26^,^27^ due to their planar geometry making the in–plane ac–field easily realized by a laser in experimental setup.

Here we consider a nearest neighbor tight–binding Hamiltonian on a honeycomb lattice with a *p*–orbital (total six states) per each site as a generic minimal model describing the 2D material at the center of interest. Because the occupation of Floquet bands remains a controversial issue^28^, we can only study the topology of a particular band gap. For simplicity, let us focus on a gap that located in the middle of the Floquet bands. In order to make our model close to actual band structures, hopping integrals are generated by a Slater–Koster method^29^ with *V*_*ppσ*_ = *t*, *V*_*ppπ*_ = −0.8*t* and onsite energy 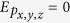. SOC is treated as a local potential by evaluating the matrix elements 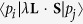 with *λ* = 0.5*t* for each site. In order to calculate the topological invariants, we implement the *n*–field method introduced Fukui *et al.*^30^,^31^. This method has been proven to provide evaluations of both *Z*_2_ and Chern topological invariants in discretized Brillouin zones accurately and efficiently. We emphasize extra time that when computing *Z*_2_ invariants for the Floquet Hamiltonian, the TR operator should be replaced by the effective TR operator 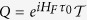 as described in this work.

**Figure 2:**
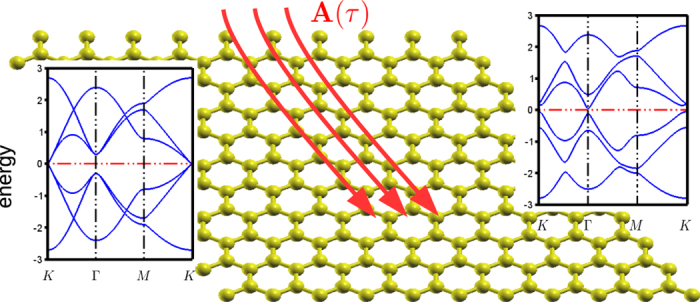
Honeycomb lattice irradiated by electric ac-field **A**(*τ*). Left inset: band structure without SOC. Right inset: band structure with SOC *λ* = 0.5*t*, *t* being a unit of energy. Note that all the bands are doubly degenerate.

**Figure 3:**
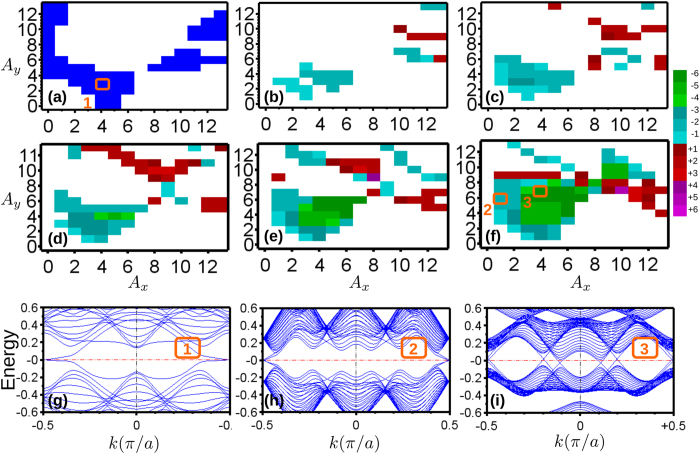
Floquet topological phase diagram. (**a**) *Z*_2_ phase diagram (blue for *Z*_2_ = 1, white for *Z*_2_ = 0) with linearly polarized ac–field [*ϕ*_*x*_, *ϕ*_*y*_] = [0, 0]. (**b**–**f**) Chern phase diagram (Chern numbers for each color are shown in the legend. White area indicates Chern number equal to zero) with polarization *ϕ*_*x*_ = 0 and *ϕ*_*y*_ = 0.1*π* ~ 0.5*π* respectively. *A*_*x*_ and *A*_*y*_ are chosen to be 0.3*n*/*a*, *n* = 0 ~ 13 (*a* is lattice constant, *ħ* = *e* = 1). (**g**–**i**) Floquet band structures of the edge states calculated in a zigzag ribbon geometry with 32 sites in the transverse direction. Edge states in (**h**) are doubly degenerate. All energies are in unit of t.

In Fig. 2, we show a cartoon of the honeycomb lattice and the band structures with and without SOC. The Dirac points at *K*-points being gapped when SOC is turned on is a general feature of most honeycomb lattice systems especially graphene. We further confirmed the SOC induced gap is topologically trival under these parameters so it behaves as a normal insulator. To study Floquet effects, we consider the reduced Floquet Hamiltonian to first order, 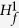, and use a rather large frequency *ω* = 6*t* (larger than the band width). The amplitudes of *A*_*x*_ and *A*_*y*_ are chosen to be 0.3*n*/*a*, *n* = 1 ~ 13 (*a* is the lattice constant) with linearly polarized field *ϕ*_*x*_ = *ϕ*_*y*_ = 0. The Floquet bands are also assumed to be half–filled as in the undriven case. Figure 3(a) shows the *Z*_2_ phase diagram. Apparently there exists a large (shown in blue) area of *Z*_2_ phase in the parameter space. To check the corresponding edge states, we have plotted the Floquet band structures of phase point 1 as a zigzag ribbon under the same ac–field. Clear edge states appear at *k* = ±*π* demonstrates the ability of tuning a normal insulator into a *Z*_2_ insulator using an ac-field.

Next, let us consider an elliptically (circularly if *A*_*x*_ = *A*_*y*_) polarized ac–field with *ϕ*_*x*_ = 0, *ϕ*_*y*_ = 0.1*π* ~ 0.5*π*. The phase diagram of the Chern numbers is shown in Fig. 3(b–f). Because it is a multiband problem (12 bands for the undriven and 36 bands for the reduced Floquet Hamiltonian), the Chern number can be much larger than ±1^32^. One can immediately find that the Chern phases are highly sensitive to the polarization angle of the ac-field. Not only the areas of non-trivial Chern phase are greatly enlarged but the values of Chern numbers are also increased when Δ*ϕ* approaches *π*/2. This is a general feature even if we change the parameters of the band structure. It suggests the more Kramer degeneracy is broken, the better is for the formation of Chern phases. In Fig. 3(i,j), we also show the edge states corresponding to the parameters of the phase point 2 and 3. The existence of 2 × 2 and 6 × 2 edge states intersect *E*_*F*_ is consistent with the Chern number *C* = −2 and *C* = −6 cases with two edges.

Finally, we estimate some physical quantities relevant to realization of such exotic electronic phases in real systems. Since the realization of topological phase transition between *Z*_2_ and Chern phases requires materials that are properly designed, we propose two rather simple applications to manipulate topological phases using the idea of controllable TRS. The first application is spin-orbit coupled graphene. It is well–known that the SOC in graphene is extremely small to be detected in experiments. Therefore the realization of quantum spin Hall effect in graphene remains a challenging issue. Recently many studies focus on the substrate or adatoms assisted SOC in graphene and have made the spin–orbit coupled graphene system possible^33^,^34^,^35^. If so, one could expect to tune the quantum spin Hall state in SOC graphene to quantum Hall state by using lasers with circular or elliptical polarization.

Let us take graphene with adatoms as an example^33^. It is predicted to have SOC induced *Z*_2_ topological gap *E*_*g*_ around 5 ~ 20 meV. To simulate this problem, we use tight–binding parameters for the *sp* states of graphene obtained by fitting to its band structure^36^,^37^ and tune the SOC to a value that in our model fits the topologically non-trival gap of 5 meV. We consider an infrared field, 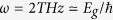. Polarization angles are chosen to be *π*/2 to maximize the Chern phases. To ensure the weak intensity approximation, we limit *A*_*x*_, *A*_*y*_ < 1(*ħ*/*A*) so that *J*_2_ effects are about two orders of magnitude smaller that *J*_1_ and can be neglected. The electric field and the corresponding laser intensity are obtained by *E*_0_ = *Aω*/*e* and 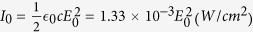 respectively. Under these conditions, we found the original *Z*_2_ phases can indeed turn into various Chern phases. It corresponds to the electric fields *E*_0_ < 1.31 × 10^5^ *V*/*cm* or the intensities *I*_0_ < 2.3 × 10^7^ *W*/*cm*^2^. This will require a rather high power about several kW in experiments. Although this power is experimentally accessible, most materials can burn out under such a strong field. Therefore searching for a material that can display both phases under lower intensities could be an interesting topic for future research.

**Figure 4:**
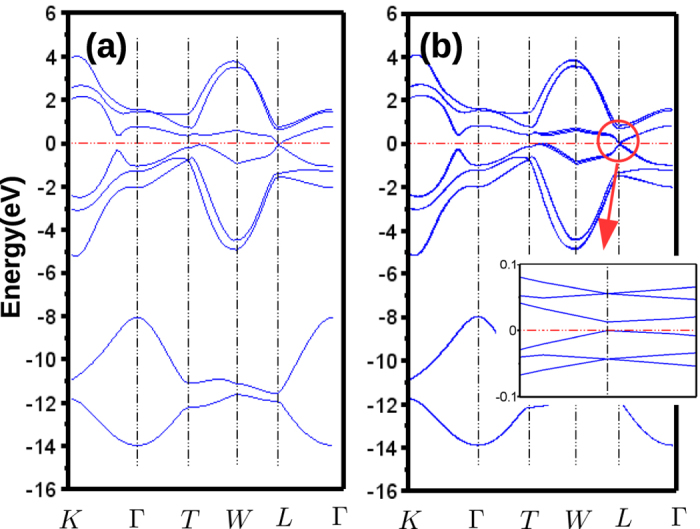
Band structure of Bi. (**a**) Static band structure (**b**) Floquet Band structure under ac–field with *ω* = 1 *meV*, [*A*_*x*_, *A*_*y*_, *A*_*z*_] = [1, 0, 1] × 10^−2^ (*ħ*/*A*). The polarization angle on all components are zero. Since *ω* is small, all bands are just slightly split. The x, y, z directions follows the same definition as ref. 38. Inset: Zoom in of the Floquet band structure around *E*_*F*_ at L-point. A gap around 15 meV is opened. *Z*_2_ calculation indicates its a topological non-trival gap with (1; 111) phase.

Another interesting application of our proposal is to generate 3D *Z*_2_ materials. Previous studies on engineering TIs using ac–fields have focused on 2D materials due to its planar structure. This makes a homogeneity of the laser field easy to achieve in experimental setup. However, if a 3D material with a size much smaller than the wavelength of the ac–field is made, it is still appropriate the consider the laser field homogeneous within the material. Obviously, this requires the field to have a macroscopic wavelength, e.g. a microwave. One of the candidates there could be crystal Bismuth. Bi is a topological trivial semi–metal but very close to its non–trival phase if its hopping parameters *V*_*ppπ*_ or *V*_*ppσ*_ are slightly perturbed^31^. It highlights the possibility of making Bi a TI by modulating the hopping parameters using the ac–field. To show this, we use a *sp* tight–binding model to describe the problem^38^. Since it has a direct gap at *L*-point with 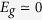, it is easy to enlarge the gap in its Floquet band structure by introducing an ac–field with a very low frequency. Apparently, it fits the microwave requirement automatically. To check whether this small gap could be topologically non–trival, we consider a linearly polarized ac–field with all *ϕ*_*i*_ = 0, *i* = *x*, *y*, *z* and *ω* = 241 *GHz* = 1 *meV* (*λ* ~ 1 *mm*) along [0, 1, 0] direction of the Bi crystal. The amplitude [*A*_*x*_, *A*_*y*_, *A*_*z*_] is set to [1, 0, 1] × 10^−2^ *ħ*/*A*. The band structures are shown in Fig. 4. One can immediately notice that a gap around 10 meV in the Floquet band structure is opened at the L point. We evaluate the *Z*_2_ invariants and obtain a non–trival (1; 111) strong TI phase. The input ac–field *E*_0_ ~ 241.8 *V*/*cm* or the intensity *I*_0_ ~ 77 *W*/*cm*^2^ requires a laser with a rather low power ~0.5 *W* accessible in experiment. Therefore, we suggest Bi could be a good choice to generate 3D *Z*_2_ TI using the Floquet effect.

## Conclusion

In summary, we have developed a framework to study TRS in Floquet Hamiltonian and used a generic tight–binding model of the honeycomb lattice relevant to several recently discovered monolyaered materials in order to demonstrate the possiblity of transitions between *Z*_2_ and Chern phases by tuning the polarization of the ac–field. Although, our discussion is based on the dynamical analogies, the physics is still very fascinating not only due to the emergence of the *Z*_2_ phase in a formally time–reversal breaking potential but also due to the possibility of manipulating contradictory topological phases in a single material. In addition, we also estimate the conditions of generating ac–field induced Chern phases and *Z*_2_ phases in real materials including spin-orbit coupled graphene and crystalline Bi. Although it is difficult to generate *Z*_2_ to Chern transition in a real system, we conclude that Bi could be a promising material to generate 3D Floquet TI. Both phenomena are hard to find in static systems but could lead us to a new physics that is unreachable in conventional solid–state matters.

## Additional Information

**How to cite this article**: Pi, S.-T. and Savrasov, S. Polarization induced *Z*_2_ and Chern topological phases in a periodically driving field. *Sci. Rep.* **6**, 22993; doi: 10.1038/srep22993 (2016).

## Supplementary Material

Supplementary Information
