# Supplementary material for: Polarization induced Z2 and Chern topological phases in a periodically driving field
Source: Sci Rep. 2016 Mar 11;6:22993. doi: 10.1038/srep22993 (PMC4786851; doi:10.1038/srep22993)
Supplement: Supplementary Information [file srep22993-s1.pdf]

Supplemental Materials: Polarization induced Z_2 and Chern topological phases in a periodically driving field

Shu-Ting Pi and Sergey Savrasov
Dept. of Physics, University of California, Davis
One Shields Ave, Davis, CA 95616

In this article, we provide proofs of two theorems.

1 Floquet Time-Reversal Symmetry

Define Floquet operator $u(T)$ and Floquet Hamiltonian H_F

$$u(T) = \mathbf{T}[e^{-i\int_0^T H(\tau)d\tau}] \equiv e^{-iH_F T} \tag{1}$$

where T is the time periodicity of the Floquet system and $\mathbf{T}$ is the time-order product. We hope to find an effective time–reversal (TR) operator $\mathcal{Q}$ for the Flouqet operator and Floquet Hamiltonian such that

$$\mathcal{Q}u(T)\mathcal{Q}^{-1} = u(-T) \tag{2}$$

and

$$\mathcal{Q}H_F(\mathbf{k})\mathcal{Q}^{-1} = H_F(-\mathbf{k}) \tag{3}$$

where $\mathcal{Q}$ is an antilinear oparator with $\mathcal{Q}^2 = -1$. We claim that if there exists an parameter τ_0 that satisfies the relation

$$\mathcal{T}H(\tau)\mathcal{T}^{-1} = H(-\tau + \tau_0) \tag{4}$$

where $\mathcal{T}$ is the conventional TR operator. Then an effective $\mathcal{Q}$ can always be defined as

$$\mathcal{Q} \equiv u(0, \tau_0)\mathcal{T} = e^{iH_F\tau_0}\mathcal{T} \tag{5}$$

In the following, we provide a proof for this theorem. (Note: Our proof is equivalent to the one shown in Ref.7 of the main article. Because we have chosen a slightly different statement, we prove it again here.)

Let us represent the conventional TR operator as the product of an unitary operator $\mathcal{S}$ (usually $e^{-i\pi\sigma_y/2}$) and the complex conjugate operator $\mathcal{K}$:

$$\mathcal{T} = \mathcal{S}\mathcal{K} \tag{6}$$

Assume there exists a parameter τ_0 such that

$$\mathcal{T}H(\tau)\mathcal{T}^{-1} = H(-\tau + \tau_0) \Longleftrightarrow \mathcal{S}H^*(\tau)\mathcal{S}^\dagger = H^\dagger(-\tau + \tau_0) \tag{7}$$

Since

$$\begin{aligned} u(T,0) = &\lim_{N\to\infty} e^{-i\Delta\tau H(T-\Delta\tau)} \times e^{-i\Delta\tau H(T-2\Delta\tau)} \times \cdots \\ &\cdots \times e^{-i\Delta\tau H(0)} \quad ; \quad \Delta\tau = T/N \end{aligned} \tag{8}$$

We have

$$\begin{aligned} \mathcal{S}u^*(T,0)\mathcal{S}^\dagger &= \lim_{N\to\infty} e^{i\Delta\tau\mathcal{S}H^*(T-\Delta\tau)\mathcal{S}^\dagger} \times \cdots \times e^{i\Delta\tau\mathcal{S}H^*(0)\mathcal{S}^\dagger} \\ &= \lim_{N\to\infty} e^{i\Delta\tau H^\dagger(\tau_0-T+\Delta\tau)} \times \cdots \times e^{i\Delta\tau H^\dagger(\tau_0)} \\ &= \lim_{N\to\infty} e^{i\Delta\tau H^\dagger(\tau_0+\Delta\tau)} \times \cdots \times e^{i\Delta\tau H^\dagger(\tau_0+T)} \\ &= u^\dagger(\tau_0+T,\tau_0) \end{aligned} \tag{9}$$

where the third equal sign uses the relation $H(\tau+T) = H(\tau)$. Therefore if we define $\mathcal{R} \equiv u(0,\tau_0)\mathcal{S}$ to shift the origin from τ_0 to 0, then an effective TR operator can be defined as $\mathcal{Q} = \mathcal{R}\mathcal{K}$

$$\begin{aligned} \mathcal{R}u^*(T,0)\mathcal{R}^\dagger &= u(0,\tau_0)\mathcal{S}u^*(\tau,0)\mathcal{S}^\dagger u^\dagger(0,\tau_0) \\ &= u(0,\tau_0)u^\dagger(\tau_0+T,\tau_0)u^\dagger(0,\tau_0) \\ &= u^\dagger(T,0) \end{aligned} \tag{10}$$

It means

$$\mathcal{Q}u(T,0)\mathcal{Q}^{-1} = u(-T,0) \tag{11}$$

and

$$\mathcal{Q}H_F(\mathbf{k})\mathcal{Q}^{-1} = H_F(-\mathbf{k}) \tag{12}$$

2 Relation to Polarization

Consider a vector potential $\mathbf{A}(\tau) = [A_x sin(\omega\tau + \phi_x), A_y sin(\omega\tau + \phi_y), A_z sin(\omega\tau + \phi_z)]$. We claim two consequences:

- If $\phi_i - \phi_j = m\pi$ $(i, j \in x, y, z,\ m \in integer)$, the Floquet time–reversal criterion: $\mathcal{T}H(\tau)\mathcal{T}^{-1} = H(-\tau + \tau_0)$ will always be satisfied.
- If $\phi_i - \phi_j = m\pi$, one can always let $\phi_i = n_i\pi$ $(n_i \in integer)$ such that $\tau_0 = 0$ and the effective TR operator can be simply expressed as $\mathcal{Q} = \mathcal{I}\mathcal{T}$.

The first theorem tells us the relation between the Floquet TR symmetry and the polarization of the ac–field. The second theorem helps us to deal with the effective TR operator in a much simpler way. In the following, we provide a proof for these two statements.

Consider the basis set of Hilbert space $\{|\alpha, \sigma\rangle\}$ where α is the label of space–related degree of freedom, e.g, sublattice, orbital, etc., and $\sigma = +/-$ is the spin index. Define time–reversal operator $\mathcal{T} = uK$ where $u = -i\sigma_y$. Then the matrix element of a time–reversal transformation applied to the Hamiltonian is given by

$$\begin{aligned}\langle\alpha\sigma|\mathcal{T}H\mathcal{T}^{-1}|\alpha^{'}\sigma^{'}\rangle &= \langle\alpha\sigma|uH^{*}u^{\dagger}|\alpha^{'}\sigma^{'}\rangle \\ &= (-1)^{[\delta_{\sigma-}+\delta_{\sigma^{'}-}]}(H^{\alpha-\sigma}_{\alpha^{'}-\sigma^{'}})^{*} \end{aligned} \tag{13}$$

If TR symmetry exists, $\mathcal{T}H\mathcal{T}^{-1} = H$, and we obtain a restriction on the matrix elements:

$$(-1)^{[\delta_{\sigma-}+\delta_{\sigma^{'}-}]}(H^{\alpha-\sigma}_{\alpha^{'}-\sigma^{'}})^{*} = H^{\alpha\sigma}_{\alpha^{'}\sigma^{'}} \tag{14}$$

For a system with an ac–field, the hopping integral is modified by $t^{\alpha\sigma}_{\alpha^{'}\sigma^{'}}(\tau) \rightarrow t^{\alpha\sigma}_{\alpha^{'}\sigma^{'}}e^{i\mathbf{A}(\tau)(\mathbf{r}_{\alpha}-\mathbf{r}_{\alpha^{'}})}$. The Floquet TR criterion $\mathcal{T}H(\tau)\mathcal{T}^{-1} = H(-\tau + \tau_0)$ requires that

$$\begin{aligned}&(-1)^{[\delta_{\sigma-}+\delta_{\sigma^{'}-}]}(t^{\alpha-\sigma}_{\alpha^{'}-\sigma^{'}})^{*}e^{-i\mathbf{A}(\tau)(\mathbf{r}_{\alpha}-\mathbf{r}_{\alpha^{'}})} \\ &= t^{\alpha\sigma}_{\alpha^{'}\sigma^{'}}e^{i\mathbf{A}(-\tau+\tau_0)(\mathbf{r}_{\alpha}-\mathbf{r}_{\alpha^{'}})}\end{aligned} \tag{15}$$

Assuming the system has TR symmetry when undriven, the hopping integrals will be canceled out and we have

$$-\mathbf{A}(\tau) = \mathbf{A}(-\tau + \tau_0) \tag{16}$$

Because

$$\mathbf{A}(\tau) = [A_x sin(\omega\tau + \phi_x), A_y sin(\omega\tau + \phi_y), A_z sin(\omega\tau + \phi_z)] \tag{17}$$

then Eq.16 means

$$
\begin{aligned}
&-(\omega\tau+\phi_i)+2n_i\pi=-\omega\tau+\omega\tau_0+\phi_x \\
&\Rightarrow \phi_i=n_i\pi-\omega\tau_0/2, \quad i\in x,y,z\ ;\ n_i\in integer
\end{aligned} \tag{18}
$$

Since τ_0 can be arbitrary real numbers, it is convenient to express the effective TR condition as

$$
\phi_i-\phi_j=m\pi, \quad m\in integer \tag{19}
$$

For an in–plane ac–field, it is to say that a linearly polarized ac–field will have the Floquet TR symmetry. Furthermore, if Eq.19 is held, we can always choose $\tau_0=0$ and $\mathcal{Q}=\mathcal{IT}$. To show this, let us shift the time frame $\tau=\tau^{'}+\tau_0/2$ and plug in the Eq.18. If so, we can get a new equation where $\phi_i=n_i\pi$ and $\tau_0^{'}=0$.
